# Protein-related hydrophobicity differences among strains belonging to *Candidozyma auris* (*Candida auris*) clades

**DOI:** 10.1128/spectrum.02897-25

**Published:** 2026-04-02

**Authors:** Samuel Rodrigues Dos Santos Junior, Piotr R. Stempinski, Arie Van Wieren, Gracen R. Gerbig, Daniel F. Q. Smith, Daniel Zamith-Miranda, Joshua D. Nosanchuk, Arturo Casadevall

**Affiliations:** 1 W. Harry Feinstone Department of Molecular Microbiology and Immunology, Johns Hopkins Bloomberg School of Public Health 25802 , Baltimore, Maryland, USA; 2 Department of Medicine (Division of Infectious Diseases) and Department of Microbiology and Immunology, Albert Einstein College of Medicine, Bronx, New York, USA; Jagiellonian University, Kraków, Poland

**Keywords:** *C. auris*, hydrophobicity, proteins, mass spectrometry, biofilms

## Abstract

In recent years, *Candidozyma auris* (*Candida auris*) has emerged as a threat to human health due to its combination of virulence and antifungal resistance. This fungal pathogen is notable for its thermotolerance and a strong attachment capability that confers environmental persistence and immune evasion. Here, we compared strains from five clades of *C. auris*: clade I = South Asia (CDC 387), clade II = East Asia (CDC 381), clade III = Africa (CDC 384), clade IV = South America (CDC 385), and clade V = Iran (CDC 1097) for their ability to regulate cell surface hydrophobicity and biofilm. The hydrophobicity varied for each *C. auris* clade, with strains 384, 385, and 1097 displaying the highest hydrophobicity levels. *C. auris* strains had variable biofilm levels when compared among clades and had lower or similar biofilm levels when compared with *Candida albicans* at different temperatures. Comparison of mass spectrometry analysis of proteolytic digestates of cells from strains with low or high hydrophobicity revealed putative surface hydrophobic proteins. These included homologs to *Candida albicans* Als3 and IFF proteins, and their contribution to hydrophobicity and biofilm formation was confirmed with gene-deficient (knockout) *C. auris* strains. We conclude that several proteins contribute to *C. auris* surface hydrophobicity.

**IMPORTANCE:**

*Candidozyma auris* (*Candida auris*) is an emerging pathogenic microorganism that is rapidly gaining attention due to outbreaks in health care facilities and its multidrug resistance. Its origin has yet to be determined, but genotypic analyses have pointed toward a simultaneous independent emergence of the different clades, possibly implicating climate change as a major factor in its recent appearance as a fungal pathogen. In 2022, the World Health Organization placed *C. auris* in the critical priority group as the second greatest fungal threat globally. Due to limited immunological and proteomic studies of *C. auris*, we sought to elucidate possible virulence mechanisms and identify leading proteins that can be targeted by immunotherapies and new drugs. Using proteomic analysis, we identified 12 lead proteins related to *C. auris* hydrophobicity and adhesion, implying that these properties are conferred by multiple proteins.

## INTRODUCTION

*Candidozyma auris* (*Candida auris*) has emerged as a global threat since it was first isolated in 2009 from the ear canal of an individual in Japan (1, 2). While subsequent retrospective analyses in clinical microbiological banks have identified several clinical isolates dating back to 1996 (1), it is clear that *C. auris* is a new fungal pathogen. *C. auris* clinical strains are frequently resistant to one or more of the three major classes of antifungal drugs (azoles, polyenes, and echinocandins) (3). *C. auris*’s capacity to form aggregates and biofilms contributes to its high persistence on surfaces and medical devices like catheters and intravenous access lines (4–6). Thermotolerance confers upon *C. auris* the ability to survive in mammalian hosts (1). In 2022, the World Health Organization classified *C. auris* as the second most important species among the critical priority fungal pathogens (7, 8).

Since 2009, several *C. auris* outbreaks have been reported around the world, and cases now occur in every continent except for Antarctica (9–12). Remarkably, the appearance of *C. auris* in different parts of the world occurred nearly simultaneously (1, 13). Groups of *C. auris* strains are genetically distinct, resulting in their classification into different clades. Clade I is usually associated with South Asian strains, clade II with East Asian strains, clade III with African strains, clade IV with South American strains, and clade V with Iranian strains. A new VI clade was described in Singapore, isolated from an individual from Bangladesh, with thousands of single-nucleotide polymorphisms differentiating the isolate from those other clades (1, 14, 15). Genetic evidence suggests that the clades evolved from a single common ancestor approximately 200 million years ago (9) and remained in the environment as saprophytic organisms. Their emergence as human pathogens has been proposed to be a result of the rise of global temperature and the use of antifungal compounds in agricultural environments, such that they simultaneously acquired thermotolerance and drug resistance, which enabled them to transition from environmental microbes to a lethal human pathogen (16), with overall mortality rates ranging from 29% to 53% (8).

*C. auris* immunological studies are in their infancy, and it remains unclear how the fungus is recognized by the immune system or what mechanisms facilitate its survival and replication ability inside macrophages (17–19). Control and elimination of the infection rely on the adaptive immune system, mainly by the induction of a Th1 and Th17 cellular immune response (20–22). The efficacy of humoral immunity against *C. auris* is not well described, but monoclonal antibodies can reduce or prevent infection in murine models (23, 24). Even though *C. auris* can evade the host immune response, outbreaks and host colonization in humans primarily arise as healthcare-acquired nosocomial infections (25, 26).

The goal of this work was to compare strains from the *C. auris* clades by assessing some characteristics, such as cell surface hydrophobicity and biofilm formation. Our results indicate key phenotypic differences between strains assigned to different *C. auris* clades, which were validated by the use of *C. auris* ALS and IFF mutants (knocked out or reconstituted).

## RESULTS

**Fig 1:**
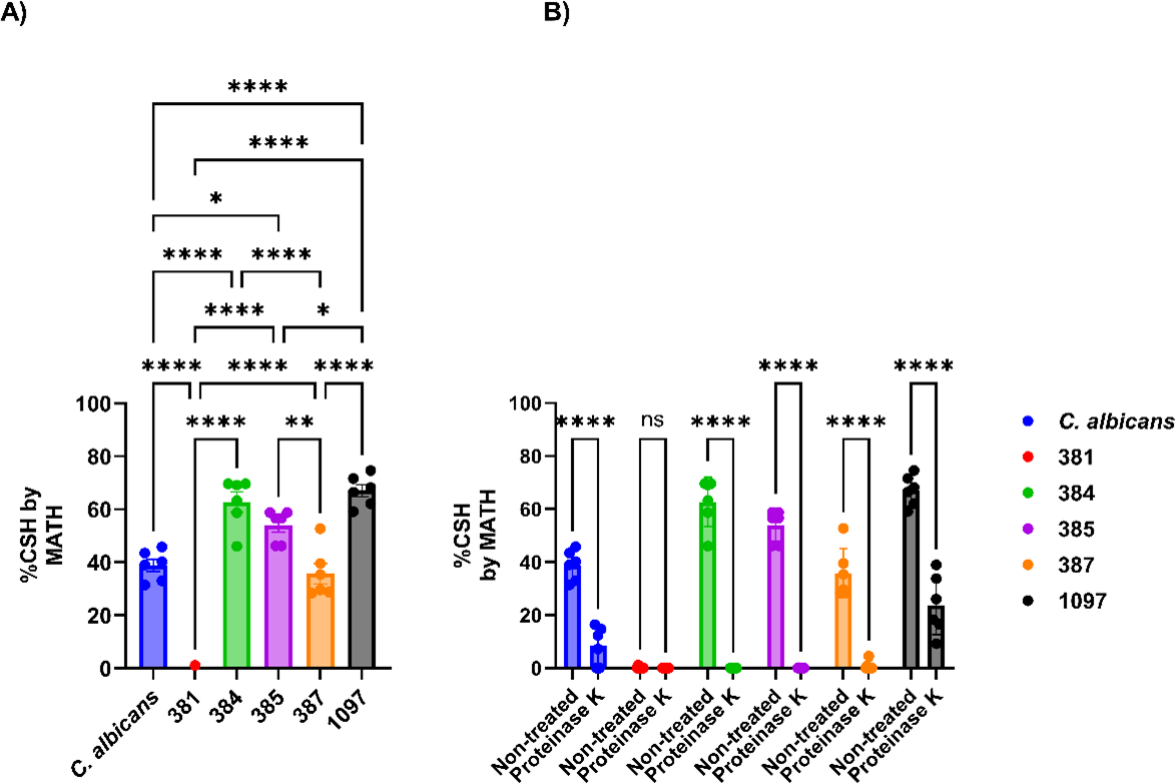
Cell surface hydrophobicity of *C. albicans* and *C. auris* strains. (**A**) Cell surface hydrophobicity differences between each *C. auris* clade. *C. albicans* strain 90028 was used as a control. (**B**) Cell surface hydrophobicity measurements after proteinase K treatment *of C. albicans* and *C. auris* strains. *C. albicans* and *C. auris* without proteinase K treatment were used as controls. *C. albicans n* = 8, Clade I = South Asia (387) *n* = 6, Clade II = East Asia (381) *n* = 6, Clade III = Africa (384) *n* = 6, Clade IV = South America (385) *n* = 6, and Clade V = Iran *n* = 6. *P < 0.05, **P < 0.01, and ****P < 0.0001. Multiple comparisons were corrected by Šídák’s multiple comparisons test (average of two biological replicates).

*Candida albicans* hydrophobicity occupied an intermediate position relative to the *C. auris* strains, being higher than strains 381, comparable to strain 387, and lower than strains 384, 385, and 1097 (Fig. 1A). Among the *C. auris* CDC strains, 384, 385, and 1097 from clades III, IV, and V, respectively, had the highest levels of hydrophobicity.

Incubation of either *C. auris* strains or *C. albicans* with proteinase K for 1 h at 37°C resulted in a significant reduction in hydrophobicity, consistent with hydrophobicity being conferred by a proteinaceous component (Fig. 1B). However, the effect of proteolytic digestion on hydrophobicity varied with the strain. Notably, *C. auris* strain 381 exhibited hydrophobicity levels close to zero in untreated and proteinase K-treated conditions, showing no significant change in its hydrophobicity profile. This inter-strain variation suggested that we may identify proteins associated with hydrophobicity through comparative analysis of the products from proteolytic digestate.

To identify the protein components of the cell wall responsible for the high hydrophobicity of the *C. auris* strains, we analyzed the protein fragments present in the supernatant fraction from cells treated with proteinase K, as well as non-treated controls. Approximately 460 proteins were identified in the supernatant of proteinase K-treated strains (Table S1). Further analysis of this protein set that was mostly present in the supernatant of proteinase K-treated strains and not in the control strains and those shared by more than one clade. This revealed 12 lead proteins that were considered candidates for conferring hydrophobicity (Table 1). Additionally, we compared the homology of the 12 selected proteins to those found in three clinically relevant *Candida* species: *C. albicans*, *C. parapsilosis*, and *C. tropicalis* (Table 1). This analysis revealed the degree of conservation of these proteins across different *Candida* species. Notably, two proteins A0A2H0ZHZ9 and A0A2H0ZP31, homologous to *C. albicans* Als3 and Gca1, were found in the highly hydrophobic *C. auris* strains, but not in the non-hydrophobic strain 381.

**TABLE 1:** BLASTp results for *Candida auris* proteins homologs in *Candida albicans, Candida parapsilosis,* and *Candida tropicalis*

Protein accessions	Protein descriptions	*C. albicans*		*C. parapsilosis*		*C. tropicalis*	
		Query cover (%)	Per. ident (%)	Query cover (%)	Per. ident (%)	Query cover (%)	Per. ident (%)
A0A2H0ZHZ9	Agglutinin-like protein	82	37.46	82	38.73	78	34.26
A0A2H0ZP31	Uncharacterized protein	96	60.06	55	32.93	91	60.69
A0A2H1A2W7	Glucan 1,3-beta-glucosidase	97	65.67	100	67.21	100	57.93
A0A2H0ZWG0	Glucan endo-1,3-beta-D-glucosidase	100	41.07	100	45.50	69	50.60
A0A2H1A533	Receptor L-domain domain-containing protein	84	60.66	84	56.23	83	57.30
A0A2H1A7S4	1,3-Beta-glucanosyltransferase	87	65.49	87	64.83	87	65.43
A0A2H0ZKA3	Hyphally-regulated cell wall protein N-terminal domain-containing protein	45	27.70	45	26.97	33	30.43
A0A2H0ZTX6	Glucan 1,3-beta-glucosidase	95	61.14	99	59.57	100	60.76
A0A2H0ZYV1	Glycosidase	83	60.10	91	56.54	99	54.99
A0A2H0ZQ75	Uncharacterized protein	58	38.64	32	38.24	57	34.51
A0A2H1A6M5	Peptidase S8/S53 domain-containing protein	85	52.12	95	46.97	94	49.62
A0A2H0ZKL3	Chitinase	70	56.92	70	55.06	68	59.87

Subsequent analyses of these proteins included an examination of their predicted localization, average hydropathicity, molecular size, and the function of their *C. albicans* homologs (Table 2). In addition, the grand average of hydropathy (GRAVY) analysis identified three proteins with scores indicative of high average hydrophobicity: A0A2H1A533, a homolog of *C. albicans* Ecm33; A0A2H0ZKA3, a homolog of *C. albicans* Iff4; and an uncharacterized protein A0A2H0ZQ75.

**TABLE 2:** Proteins identified in the supernatant of *Candidozyma auris* exposed to proteinase K treatment

Protein accessions	Protein descriptions	Predicted localization	GRAVY	Size (kDa)	*C. albicans* homolog	Function in homologs
A0A2H0ZHZ9	Agglutinin-like protein	Cell membrane, secreted	−0.091	183.7	Als3, Als4	Adhesion of the fungal cell to host and abiotic surfaces (27, 28)
A0A2H0ZP31	Uncharacterized protein	Secreted	−0.361	112.3	Gca1, Gca2	Promote biofilm production by hydrolytic release of soluble β-1,3-glucan fragments to the matrix (29)
A0A2H1A2W7	Glucan 1,3-beta-glucosidase	Cell wall, secreted	−0.252	33.8	Bgl2	Cell wall biosynthesis and virulence, delivery of beta-1,3-glucan to the biofilm matrix (30)
A0A2H0ZWG0	Glucan endo-1,3-beta-D-glucosidase	Secreted	−0.199	114.9	Eng1	Eng1 and Xog1 play a role in fungal adhesion to host cells, regulated β-glucan exposure in yeast (31, 32)
A0A2H1A533	Receptor L-domain domain-containing protein	Cell membrane	0.019	43.9	Ecm33	Involved in fungal cell wall integrity, and together with Bgl2 and Als1, important for cell adhesion (33, 34)
A0A2H1A7S4	1,3-Beta-glucanosyltransferase	Cell membrane	−0.227	58.9	Phr2	Construction and cross-linking of beta-1,3 and beta-1,6-glucans within the fungal cell wall (35)
A0A2H0ZKA3	Hyphally-regulated cell wall protein	Secreted	0.018	71.3	Iff4	IFF4 encodes a protein that helps the fungus adhere to host cells and surfaces (36, 37)
A0A2H0ZTX6	Glucan 1,3-beta-glucosidase	Secreted	−0.538	47.8	Xog1	Promote the adherence of *C. albicans* cells to host epithelial cells (31, 38)
A0A2H0ZYV1	Glycosidase	Secreted, plasma membrane	−0.46	49.6	Utr2	Helps with cell wall assembly and regeneration. It also plays a role in filamentation and adherence (39)
A0A2H0ZQ75	Uncharacterized protein	Secreted	0.065	60.2	Cell wall protein homolog	–^*a*^
A0A2H1A6M5	Peptidase S8/S53	Secreted	−0.12	43.7	Uncharacterized protein	–^*a*^
A0A2H0ZKL3	Chitinase	Secreted	−0.313	47.2	Cht3	Cell wall remodeling, cell separation (40)

^*a*^ –, no homologous function found.

**Fig 2:**
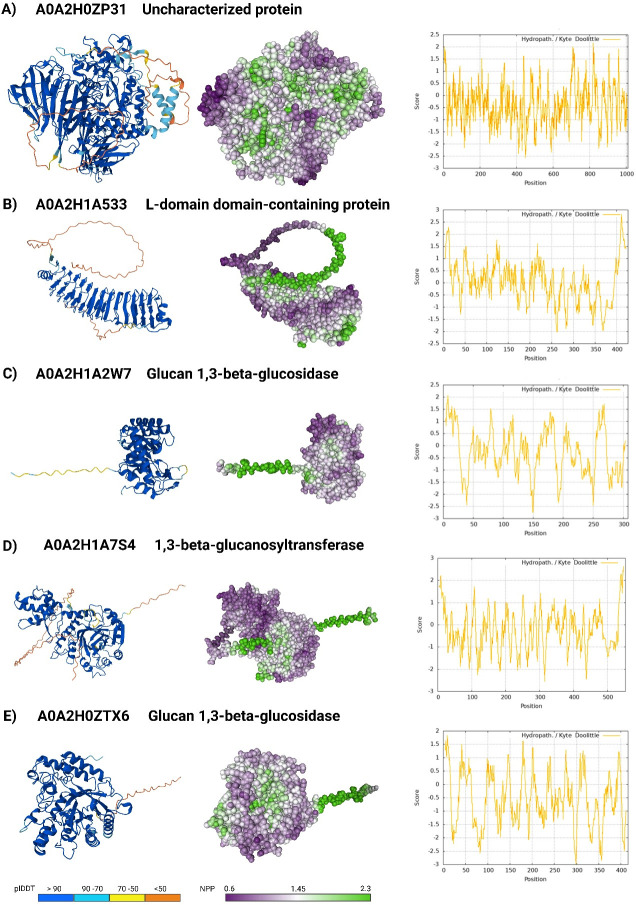
Visualization of 3D protein structure and surface hydrophobicity patches. The figure shows 3D models of protein visualization and predicted areas of relative protein hydrophobicity for selected proteins with a high confidence of Alphafold3 prediction (pTm > 0.8). Predicted structure visualization was created for (**A**) A0A2H0ZP31, (**B**) A0A2H1A533, (**C**) A0A2H1A2W7, (**D**) A0A2H1A7S4, and (**E**) A0A2H0ZTX6. The colors in the structures represent the prediction confidence score: dark blue (very high), light blue (confident), yellow (low), and orange (very low). The areas with a high ratio of non-polar to polar (NPP) values are indicated with purple, and areas with low NPP values are marked with green. A hydrophobicity profile plot shows the distribution.

The homology analysis of these 12 hydrophobicity-associated proteins revealed varying degrees of conservation across different *Candida* species, with only two proteins showing significant homology to human proteins. The peptidase S8/S53 domain-containing protein A0A2H1A6M5 shares 35.83% identity with human protein convertase subtilisin/kexin type 9, while the uncharacterized protein homolog of Gca1 shows 31.63% identity to human lysosomal alpha-glucosidase. To gain insight into how the structure of these 12 proteins might contribute to *C. auris* hydrophobicity, their putative structures were generated using Alphafold3 and then analyzed using the Protein-Sol server to visualize and identify hydrophobic surface patches (Fig. 2; Fig. S1). This approach revealed multiple potential surface hydrophobic patches in the protein set. Five proteins had their prediction pTm scores approximately ≥0.8 (Fig. 2), indicating they represent confident, high-quality predictions, while the remaining seven proteins had lower pTm scores (Fig. S1).

**Fig 3:**
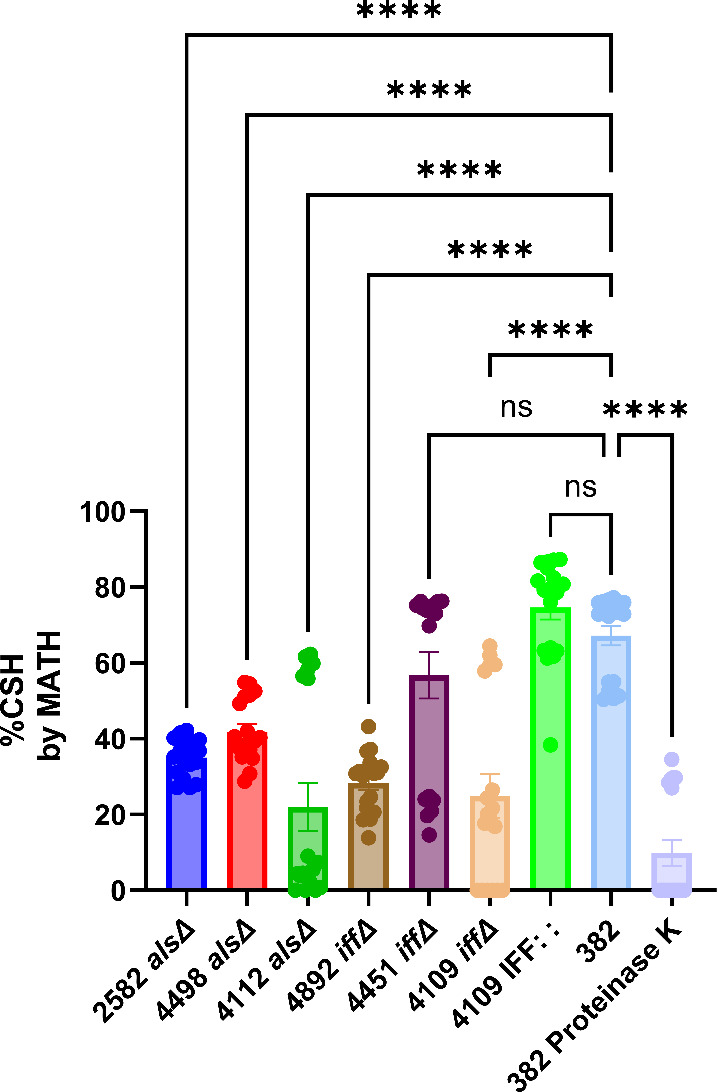
Cell surface hydrophobicity of *C. auris* strain 382 ALS and IFF adhesins family knockout. Cell surface hydrophobicity varied according to knocked-out protein, except for the 4451 *iffΔ* (A0A2H0ZKA3). The 4112 *alsΔ* (A0A2H0ZHZ9) knocked out protein showed the lowest hydrophobicity level. The recovered 4109 IFF : : protein had its hydrophobicity restored to the same level as the parental strain (382). **** = *P* < 0.0001 (*t*-test and or analysis of variance [ANOVA]). ns = not significant. Multiple comparisons were corrected by Šídák’s multiple comparisons test. *n* = 18 (average of three biological replicates).

To validate and possibly confirm the insights gleaned from the proteomic and structural analysis, we obtained *C. auris* strains deficient (knockout) for genes in the ALS and IFF families in the strain background 382 and evaluated them from cell surface hydrophobicity and biofilm formation. Two strains deficient in 4112 *alsΔ* (A0A2H0ZHZ9) and 4451 *iffΔ* (A0A2H0ZKA3) were present in our list of selected proteins (Tables 1 and 2). In addition, we studied strains deficient in 2582 *alsΔ,* 4498 *alsΔ,* 4892 *iffΔ,* and 4109 *iffΔ,* which were detected by our proteomic analysis but failed to reach our selection criteria (Table S1). Most of the strains deficient in these proteins manifested reduced hydrophobicity except for the 4451 *iffΔ* (Fig. 3), strongly suggesting that this property emerges from the expression of several proteins. Reconstitution of 4109 IFF : : protein had its hydrophobicity restored to the parental strain (382) level.

**Fig 4:**
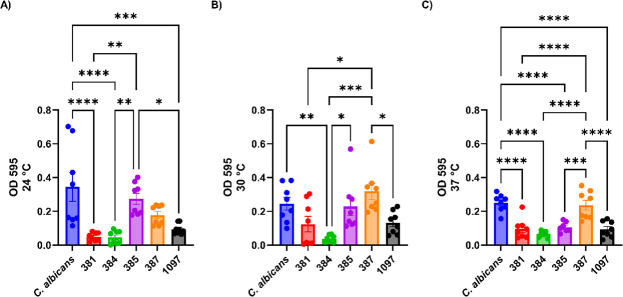
Biofilm production differences between the different clades of *C. auris* in different growth temperatures. (**A**) Biofilm formation at 24°C, (**B**) biofilm formation at 30°C, and (**C**) biofilm formation at 37°C. Clade I = South Asia (387) *n* = 8, Clade II = East Asia (381) *n* = 8, Clade III = Africa (384) *n* = 8, Clade IV = South America (385) *n* = 8, and Clade V = Iran (1097) *n* = 8. *C. albicans* strain 90028 was used as a control, *n* = 8. *P < 0.05, **P < 0.01, ***P < 0.001, **** = *P* < 0.0001 (*t*-test and or ANOVA). Multiple comparisons were corrected by Šídák’s multiple comparisons test (average of two biological replicates).

**Fig 5:**
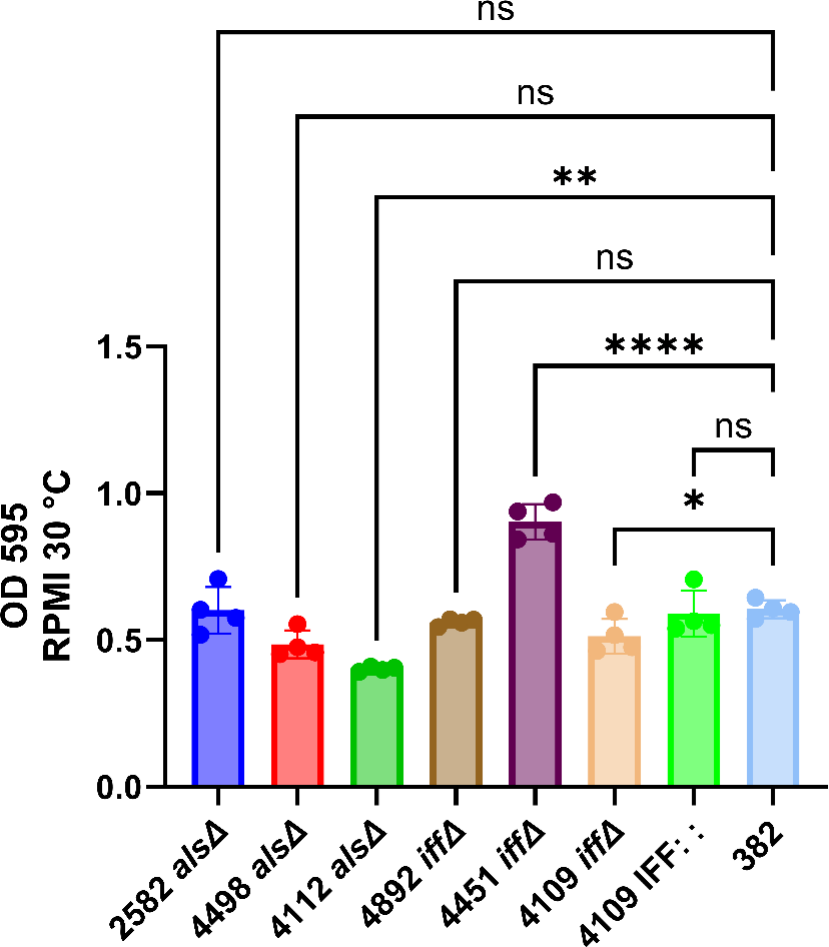
Biofilm production of *C. auris* strain 382 ALS and IFF adhesins family knockouts. Biofilm formation was protein-dependent. Two proteins were directly related to the reduction of biofilm formation [4112 *alsΔ* (A0A2H0ZHZ9) and 4109 *iffΔ*], and the knockout for the 4451 *iffΔ* (A0A2H0ZKA3) protein had its biofilm formation increased. The recovered 4109 IFF : : protein had its biofilm formation restored to the same level as the parental strain (382). * = *P* < 0.05, ** = *P* < 0.01, **** = *P* < 0.0001 (*t*-test and or ANOVA). ns = not significant. Multiple comparisons were corrected by Šídák’s multiple comparisons test. *n* = 4 (average of two biological replicates)*.*

*C. albicans* produced more biofilm than *C. auris* strains 381, 384, and 1097 in all three evaluated temperatures (Fig. 4). Biofilm levels varied from non-significant at 24°C (Fig. 4A) and 30°C (Fig. 4B) to a significant variation at 37°C (Fig. 4C) for strain 385 when compared with *C. albicans*. Strain 387 had no significant variation in all evaluated temperatures when compared with *C. albicans* (Fig. 4). Biofilm production by the gene-deficient strains varied based on the missing protein (Fig. 5). Deletion of most proteins had no influence on biofilm production after 72 h in RPMI media, but strains deficient in proteins 4112 and 4109 (4112 *alsΔ* and 4109 *iffΔ*) manifested a significant decrease in biofilm formation, while the reconstituted strain 4109 IFF had biofilm formation similar to the parental strain (382). The strain deficient in protein 4451 (4451 *iffΔ*) had increased biofilm formation capability relative to the parental strain (382).

## DISCUSSION

In this study, we compared the hydrophobicity levels and biofilm formation between strains from five clades of the opportunistic fungi *C. auris*, including *C. auris alsΔ* and *iffΔ* deficient strain knockouts. We used *C. albicans* as a control, given that it has been extensively studied and provides a point for comparison when considering the results with *C. auris*. While this study was in progress, a sixth clade was identified in Singapore, from a patient transferred from Bangladesh (14, 15, 41), but it was not included in this study. Due to our comparative analysis of the identified proteins (selected proteins were present in more than one strain and only in the proteinase K-treated cells) and the high cost of the mass spectrometry experiments and analysis, we decided to select single strains from each clade. Nevertheless, biofilm formation or hydrophobicity differences have not been subject to inter-clade analysis (36, 42).

Hydrophobicity, aggregation, and biofilm formation are each associated with the persistence of *C. auris* on surfaces, resistance to chlorine agents, and escape from the immune system (4, 5, 43).

The hydrophobicity levels for strains from different clades varied significantly. Treatment of *C. a*uris and *C. albicans* with proteinase K significantly reduced hydrophobicity levels for all strains except for *C. auris* 381, implying that this property originated from a proteinaceous component. Biofilm production by the strains from the five *C. auris* clades was evaluated at three different temperatures, with *C. albicans* as a control. Most of the *C. auris* strains produced less or comparable amounts of biofilm as *C. albicans* at 24°C, 30°C, and 37°C. Intra-clade comparisons among the *C. auris* strains showed significant variability at all evaluated temperatures. The cell walls of *C. a*uris and *C. albicans* are covered with mannoproteins, adhesins, and other uncharacterized proteins (17, 44). Disruption of surface proteins led to the reduction of hydrophobicity and loss of the aggregative status of some *C. auris* clades (5, 17, 27, 45)*.* Differences in the magnitude of the hydrophobicity reduction between the strains after proteinase K treatment imply strain-related differences in surface protein contribution to this parameter.

Analysis of the proteolytic digestate using mass spectrometry identified several proteins potentially involved in the hydrophobicity of *C. auris* cells. Among proteins identified exclusively in the supernatant from proteinase K-treated *C. auris,* only two proteins were identified mostly in highly hydrophobic strains (384, 385, and 1097). Those two proteins are homologs of *C. albicans* Als3 and Gca1. Als3 is an adhesin involved in virulence, especially in fungal-host cell adhesion and biofilm formation (46–48). The presence of Als3 homolog in highly hydrophobic strains of *C. auris* suggests a potential role in cell adhesion (46–48). *C. albicans* glucoamylases Gca1 and Cca2 have been recognized for their role in biofilm matrix production (49, 50). The identification of highly hydrophobic proteins such as Ecm33, Iff4 homologs, and the uncharacterized protein A0A2H0ZQ75 indicates a potentially important component of the cell surface hydrophobicity complex. Both Ecm33 and Iff4 have been reported to play a crucial role in cell adherence to epithelial cells and inorganic surfaces (34, 37, 44, 51).

Given the results of the proteomic analysis and the high likelihood that ALS family proteins were involved in surface hydrophobicity from prior reports in *C. albicans* (52, 53), we tested a panel of *C. auris* strains deficient in these proteins. Strains deficient in ALS family protein 4112 and IFF family protein 4109 exhibited relatively large reductions in hydrophobicity related to the parent strains that, for the 4109 deficient strains, were restored by gene complementation. A similar result was observed by Santana et al. for the IFF 4109 protein knockout and their restored counterpart when evaluated for cell surface hydrophobicity (CSH) measured by the microbial adhesion to hydrocarbons (MATH) assay (36). ALS and IFF proteins were first described in *C. albicans*, but homologs are also present in non-albicans *Candida* species such as *C. dubliniensis*, *C. tropicalis, C. auris,* and *C. parapsilosis* complex (*Candida parapsilosis, C. orthopsilosis, and C. metapsilosis)* (54–56), and their function has been associated with cell wall structure and integrity, binding to keratinocytes, polystyrene, surface attachment, skin colonization, and virulence and pathogenicity (36, 54, 57).

Some of the proteins represented in the knock-out strains were either not detected in our proteomic analysis, or their peptides did not reach our selection criteria threshold, which could reflect differences in proteins present in each clade/strain, and even proteins from the same family. For example, comparison of the strains with deleted 4451 IFF and 4109 IFF genes manifested different hydrophobicity and biofilm formation. With regard to the absence of some proteins from the proteolytic digestate, we cannot distinguish between the possibilities that the proteinase K treatment was not enough to separate the proteins from the cell surface or that these proteins are not present on the cell surface.

The utilization of Alphafold3, Protein-sol patches tool, and ProtScale software to generate structural prediction models for these proteins provided additional insights into their potential hydrophobic properties. We were unable to analyze the hydrophobicity patches for the protein A0A2H0ZHZ9 due to its low pTm score. The uncharacterized protein A0A2H0ZP31 exhibited a high non-polar/polar (NPP) ratio in its predicted structure, a feature often associated with hydrophobic interactions. Notably, our analysis revealed that most of the proteins detected in the surface protein component of highly hydrophobic *C. auris* cells present a significant homology to *C. albicans* proteins involved in cell wall maintenance and adherence, such as Xog1, Eng1, Ecm33, Iff4, Utr2, Phr2, Bgl2, and Cht3 (30, 31, 35, 39, 40). These findings indicate the conserved mechanisms responsible for cellular adherence across these fungal species and provide a potential explanation of the molecular factors contributing to *C. auris* hydrophobicity and adherence.

In summary, our work demonstrated that strains from each of the five *C. auris* clades display different hydrophobicity levels. Comparison of proteinase digestates from high and low hydrophobicity strains identified several protein candidates that contribute to surface hydrophobicity and implied that multiple proteins contributed to this property. The clade-related variation in our results highlights the need to include representatives of each clade in biological studies to understand the range inherent in this species.

## MATERIALS AND METHODS

### *C. auris* and *C. albicans* strains and media growth conditions

*C. auris* strains clade I = South Asia (387 and 382) isolated from blood and skin, respectively, clade II = East Asia (381) isolated from ear, clade III = Africa (384) isolated from blood, clade IV = South America (385) isolated from blood and clade V = Iran (1097) isolated from ear, was acquired from the Centers for Disease Control and Prevention and the Food and Drug Administration Antibiotic Resistance Isolate Bank (AR Bank), only one strain per clade was used. *C. albicans* strain 90028 was acquired from ATCC (Manassas, VA, US) and was utilized as a control strain for all experiments.

All strains were cultivated in Sabouraud Dextrose broth (SDB) or Sabouraud Dextrose agar (SDA) (BD DIFCO—Franklin Lakes, NJ, US). The liquid cultures were incubated at 30°C in a spinning wheel at 150 rpm for 24 h prior to use in experiments. The cultures grown in solid media were incubated at 30°C. Cultures were maintained in glycerol (50%) stocks at −80°C.

### Hydrophobicity

CSH was measured by the MATH assay (58). The *C. auris and C. albicans* were grown in SDB for 24 h at 30°C in a spinning wheel at 150 rpm. After growth, the *C. au*ris and *C. albica*ns cells were washed twice with PBS at 3,000 rpm for 5 min. The *C. au*ris or *C. albica*ns cells were then resuspended in 3 mL of PBS at an estimated optical density (OD_600_) of 0.2 to 0.4, vortexed with 400 µL hexadecane (Sigma-Aldrich) for 60 s, and left to sit at room temperature (24°C) for 2 min to allow the layers to separate. After the rise of the hydrophobic layer, the bottom layer (aqueous layer) was transferred to a 96-well plate, and the OD_600_ was measured in the SpectraMAX 340 Tunable Microplate Reader (Molecular Devices Ltd.). Due to their hydrophobicity, a proportion of cells enter the hydrophobic layer, resulting in a decreased absorbance in the aqueous layer. CSH was estimated as


%CSH=[(A0−A1)/A0]×100.

Where A0 = absorbance of cells before the addition of the hexadecane and A1 = absorbance of the aqueous layer after cells entered the hydrophobic layer.

To evaluate the contribution of cell wall proteins to *C. au*ris and *C. albica*ns hydrophobicity, a *C. au*ris or *C. albica*ns cell suspension at an estimated optical density (OD_600_) of 0.2 to 0.4 was incubated in 3 mL of PBS with 50 µg/mL of proteinase K (New England Biolabs—Ipswich, MA, US) for 1 h at 37°C. *C. au*ris and *C. albica*ns cells incubated in the same conditions, but without proteinase K (non-treated), were used as controls. Hydrophobicity was measured as described above.

### Biofilm formation

Biofilm formation was evaluated using a crystal violet assay (Sigma-Aldrich) (59). The fungi were pre-cultured in SDB for 24 h at 30°C in a spinning wheel at 150 rpm. After growth, *C. au*ris cells were washed twice with PBS at 3,000 rpm for 5 min. *C. au*ris cells were then added to a 96-well polystyrene untreated plate at an OD_600_ of 0.2, or equivalent to ~2 × 10^6^ cells/mL for 72 h at 30°C in RPMI-1640 (Gibco, Waltham, MA, USA) without agitation. After 72 h, the wells were washed twice with 200 µL of PBS and then air-dried for 45 min. After the drying process, 110 μL of 0.4% crystal violet was added to the wells, and the plates were incubated at room temperature (24°C) for 45 min. After the incubation, the plate was washed four times with 350 μL of sterile distilled water. Then, 200 μL of 95% ethanol was added to the wells, and the plate was incubated at room temperature (24°C) for 45 min. After incubation, 100 μL of ethanol plus crystal violet solution was transferred to a new 96-well plate, and the amount of the crystal violet stain in the ethanol was assessed by measuring the absorbance of the solution with the microtiter plate reader SpectraMAX 340 Tunable Microplate Reader (Molecular Devices Ltd.) at 595 nm.

### Mass spectrometry analysis

*C. auris* was grown in SDB for 24 h at 30°C in a spinning wheel at 150 rpm. After growth, *C. au*r*is* cells were washed twice with PBS at 3,000 rpm for 5 min. To investigate the identity of hydrophobic proteins in the yeast cell wall, a suspension at an estimated optical density (OD_600_) of 0.2 to 0.4 was incubated in 3 mL of PBS with 50 µg/mL of proteinase K (New England Biolabs—Ipswich, MA, US) for 1 h at 37°C. The proteinase K was inactivated by incubating the yeast cells for 10 min at 95°C. The yeast cells were then separated from the proteolytic digestate using a Spin-X 0.22 µm tube (Costar-Salt Lake City, UT, US). The supernatant was sent to the Mass Spectrometry and Proteomics Core at the Johns Hopkins School of Medicine (Johns Hopkins University, The Center for Proteomics Discovery). Samples were analyzed by reverse-phase chromatography tandem mass spectrometry on a Vanquish NEO nanoLC system HPLC interfaced with a Thermo Exploris 480 mass spectrometer (Thermo Fisher Scientific). The samples were injected using a trap and elute setup onto a 75 um × 25 cm column house packed with ReproSil 100A, 2.4 um particles (Dr. Maisch—Ammerbuch-Entringen, Germany). The peptides were separated at 300 nL/min over a 90 min gradient running from 0.1% formic acid to 95% acetonitrile. The peptides were ionized into the mass spectrometer with a post-column emitter setup running at 2,200 V. The data were searched with a tolerance. The samples were run at 120K MS and 30K MS resolution with a 3 s cycle time. Peptides were searched against the UniProt database (https://www.uniprot.org/) for *C. auris*, using Sequest within Proteome Discoverer Software 3.1 (Thermo Fisher Scientific) under the following parameters: 10 ppm precursor tolerance, 0.02 Daltons fragment ion tolerance, high peptides at 1% false discovery rate, and medium peptides at 5%. *C. au*ris yeast cells incubated in the same conditions, but without proteinase K (non-treated), were used as a control. No other proteolytic digestion was performed (e.g., trypsinization).

### Mass spectrometry data processing

The raw mass spectrometry data were processed and organized to corresponding proteins from a set of selected *C. auris* reference strains. These proteins were categorized based on their presence across the tested samples. To identify proteins potentially involved in the hydrophobicity of *C. auris* strains, we focused on those exhibiting an increased abundance in samples treated with Proteinase K, which is known to cleave surface-exposed proteins. Proteins were considered for further analysis if their presence was mostly found in the Proteinase K-treated samples compared to untreated controls. To finalize the selection, we applied additional criteria: only proteins that showed increased abundance in at least two out of four hydrophobic *C. auris* strains were included in the final list. This approach ensured that the selected proteins were not only responsive to Proteinase K treatment but also consistent across a range of hydrophobic *C. auris* strains, highlighting those most likely to be involved in the strain’s hydrophobicity.

### *In silico* analysis of selected *C. auris* proteins

Molecular weight (size) of each protein was also obtained from UniProt (https://www.uniprot.org/) (60).

The GRAVY values, reflecting the hydrophobicity of the proteins, were calculated using the ProtParam (https://web.expasy.org/protparam/) (61).

The BLASTp analysis was performed in UniProt (https://www.uniprot.org/) (60) to identify homologs of the *C. auris* proteins across three *Candida* species: *Candida albicans*, *Candida parapsilosis*, and *Candida tropicalis*. For each *C. auris* protein, the corresponding sequence was used as the query in BLASTp searches. The results were analyzed to identify the best matching homologs in each species, and for each match, the query cover and percentage identity were recorded.

Protein structures were modeled using full-length protein sequences by using the Alphafold3 Server (https://alphafoldserver.com/) (62) using fasta files obtained from Uniprot. Ranking score, pLDDT, and iptm scores were assessed to determine the accuracy of the predicted structure. Only models of the highest confidence, based on both pLDDT and pAE values, were utilized.

Surface hydrophobicity of the Alphafold3 structures was determined using the Protein-sol patches tool (https://protein-sol.manchester.ac.uk/patches) (63). Photos were taken from angles displaying the greatest number of hydrophobic patches.

The ProtScale (https://web.expasy.org/protscale/) tool, based on amino acid scale: hydropathicity (64) was used to generate Kyte and Doolittle plots displaying the hydrophobicity or hydrophilicity of each amino acid in sequences of full-length proteins.

### Influence of ALS and IFF family proteins on cell surface hydrophobicity and biofilm formation

*C. auris* (382 AR Bank background) ALS and IFF adhesins family knockout (ko) (2582 *alsΔ*, 4498 *alsΔ,* 4112 *alsΔ,* 4892 *iffΔ,* 4451 *iffΔ, and* 4109 *iffΔ*) and reconstituted (4109 IFF) strains were kindly provided by Dr. Teresa O’Meara from the Department of Microbiology and Immunology at the University of Michigan (36, 57). Strains were cultivated in SDB or SDA (BD DIFCO—Franklin Lakes, NJ, US). The liquid cultures were incubated at 30°C in a spinning wheel at 150 rpm for 24 h prior to use in experiments. The cultures grown in solid media were incubated at 30°C. Cultures were maintained in glycerol stocks at −80°C. Cell surface hydrophobicity and biofilm were evaluated as described previously.

### Statistical analysis

Analysis of variance (ANOVA) for multiple comparisons (all groups against all groups or all groups against the control) or student *t*-test (pairwise comparison) was performed, followed by Tukey or Dunnett post-tests, respectively, using Graph Pad Prism 10. Multiple comparisons were corrected by Šídák’s multiple comparisons test. *P* values were considered significant when *P* ≤ 0.05, and error bars were used representing the standard error of the mean (SEM).

## Data Availability

The mass spectrometry proteomics data have been deposited to the ProteomeXchange Consortium via the PRIDE (65) partner repository with the data set identifier PXD074750.

## SUPPLEMENTAL MATERIAL

Table S1 spectrum.02897-25-s0001.xlsx Proteins identified in the supernatant of *Candidozyma auris* exposed to proteinase K treatment.

Fig. S1 spectrum.02897-25-s0002.docx Visualization of 3D protein structure and surface hydrophobicity patches.

ASM does not own the copyrights to Supplemental Material that may be linked to, or accessed through, an article. The authors have granted ASM a non-exclusive, world-wide license to publish the Supplemental Material files. Please contact the corresponding author directly for reuse.
