# Supplementary material for: Protein-related hydrophobicity differences among strains belonging to Candidozyma auris (Candida auris) clades
Source: Microbiol Spectr. 2026 Apr 2;14(5):e02897-25. doi: 10.1128/spectrum.02897-25 (PMC13141915; doi:10.1128/spectrum.02897-25)
Supplement: Fig. S1 — Visualization of 3D protein structure and surface hydrophobicity patches. [file spectrum.02897-25-s0002.docx]

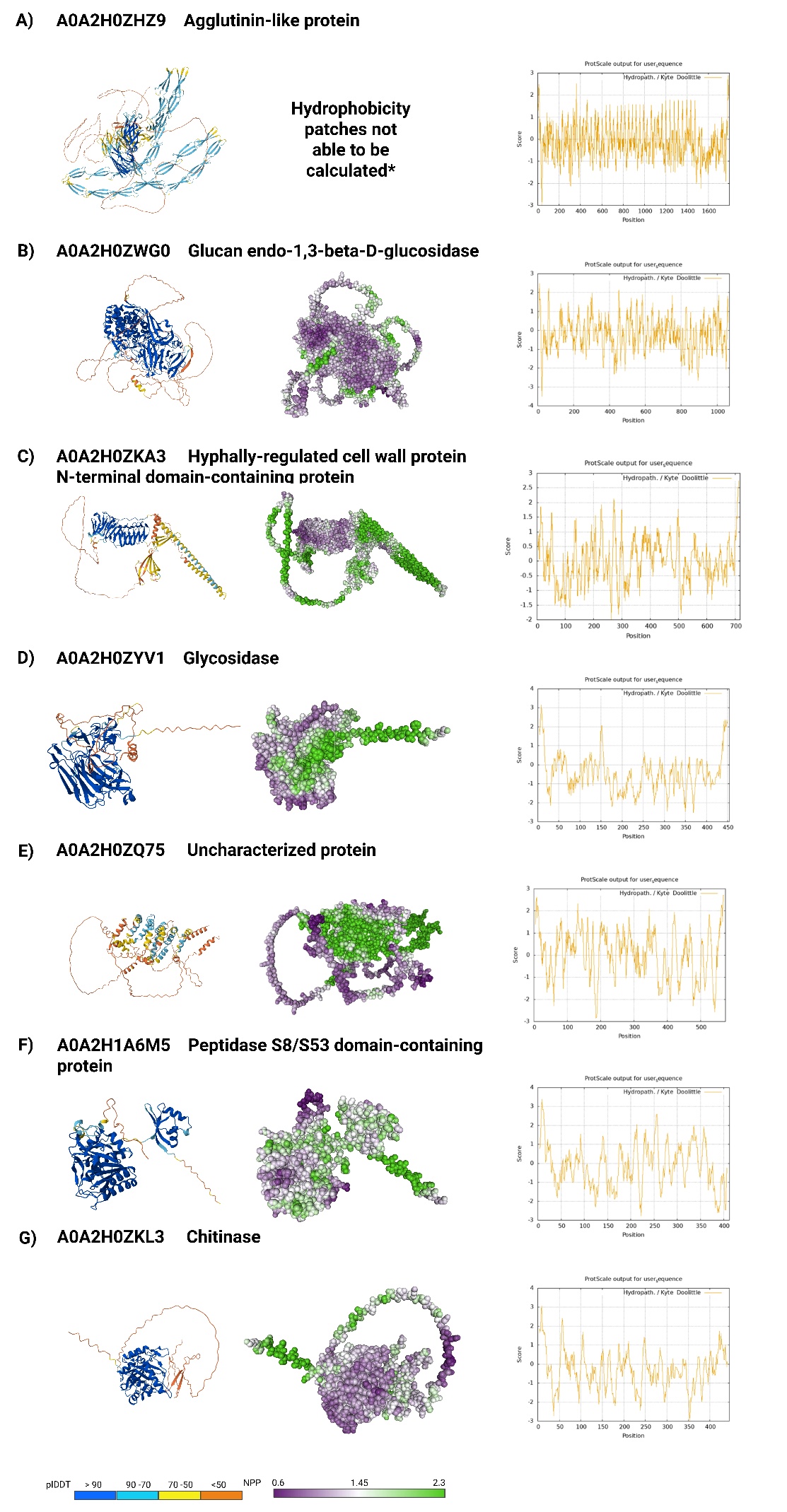


Fig. S1. Visualization of 3D protein structure and surface hydrophobicity patches. The Figure shows 3D models of protein visualization and predicted areas of relative protein hydrophobicity for selected proteins with a low confidence of Alphafold3 prediction (pTm < 0.8). Predicted structure visualization were created for A) A0A2H0ZHZ9, B) A0A2H0ZWG0, C) A0A2H0ZKA3, D) A0A2H0ZYV1, E) A0A2H0ZQ75, F) A0A2H1A6M5 and G) A0A2H0ZKL3. The colors in the structures represent the prediction confidence score: dark blue (very high), light blue (confident), yellow (low) and orange (very low). The areas with a high ratio of non-polar to polar (NPP) values are indicated with purple, and areas with low NPP values are marked with green. A hydrophobicity profile plot shows the distribution.* Protein-Sol server failed to calculate the hydrophobicity patches.

Tab. S1. Proteins Identified in the Supernatant of *Candidozyma auris* Exposed to Proteinase K Treatment.
